# Software Engineering Frameworks Used for Serious Games Development in Physical Rehabilitation: Systematic Review

**DOI:** 10.2196/25831

**Published:** 2021-11-11

**Authors:** Jorge Fernando Ambros-Antemate, María Del Pilar Beristain-Colorado, Marciano Vargas-Treviño, Jaime Gutiérrez-Gutiérrez, Pedro Antonio Hernández-Cruz, Itandehui Belem Gallegos-Velasco, Adriana Moreno-Rodríguez

**Affiliations:** 1 Doctorado en Biociencias Facultad de Medicina y Cirugía Universidad Autónoma “Benito Juárez” de Oaxaca Oaxaca de Juárez Mexico; 2 Escuela de Sistemas Biológicos e Innovación Tecnológica Universidad Autónoma “Benito Juárez” de Oaxaca Oaxaca de Juárez Mexico; 3 Laboratorio de genómica y proteómica Centro de investigación UNAM-UABJO Facultad de Medicina y Cirugía UABJO Oaxaca de Juárez Mexico; 4 Facultad de Ciencias Químicas Universidad Autónoma “Benito Juárez” de Oaxaca Oaxaca de Juárez Mexico

**Keywords:** serious game, physical rehabilitation, framework, methodology

## Abstract

**Background:**

Serious games are a support in the rehabilitation process for treating people with physical disabilities. However, many of these serious games are not adapted to the patient’s needs because they are not developed with a software engineering framework with a set of activities, actions, and tasks that must be executed when creating a software product. Better serious games for rehabilitation will be developed if the patient and therapist requirements are identified, the development is planned, and system improvements and feedback are involved. The goal is that the serious game must offer a more attractive environment, while maintaining patient interest in the rehabilitation process.

**Objective:**

This paper submits the results of a systematic review of serious games in physical rehabilitation identifying the benefits of using a software engineering framework.

**Methods:**

A systematic research was conducted using PubMed, PEDro (Physiotherapy Evidence Database), IEEE Xplore, ScienceDirect, ACM Digital Library, Mary Ann Liebert, Taylor & Francis Online, Wiley Online Library, and Springer databases. The initial search resulted in 701 papers. After assessing the results according to the inclusion criteria, 83 papers were selected for this study.

**Results:**

From the 83 papers reviewed, 8 used a software engineering framework for its development. Most of them focused their efforts on 1 or more aspects, such as data acquisition and processing, game levels, motivation, therapist supervision.

**Conclusions:**

This systematic review proves that most of the serious games do not use a software engineering framework for their development. As a result, development systems overlook several aspects and do not have a standardized process, eventually omitting important implementation aspects, which impact the patient’s recovery time.

## Introduction

### Overview

According to the World Health Organization, over 1 billion people have some form of disability [1], with up to 200 million people having loss or decrease in movement, which limits their ability to perform activities of daily living. To overcome it, they must undergo a rehabilitation program to gradually regain movement and consequently, improve their quality of life.

However, the traditional rehabilitation process is often slow and presents problems such as lack of motivation, boredom, and others; as a result, many patients consider the exercises stressful, and therefore abandon the therapy [2].

To avoid these situations, new ways of conventional therapy support have been used in recent years, such as medicinal treatments, robotics, video games (known as serious games), and others [3], which have contributed to faster rehabilitation when performing exercises in a fun way, allowing the patients to forget their conditions and concentrate on the game.

For this reason, new interaction modes, such as serious games [4], have the potential to provide more attractive, motivating, and enriching experiences for patients who suffer from decreases in movement. Currently, serious game–based physical rehabilitation is an area of research in constant evolution, and therefore, there is the need for developing guidelines adapted from other research fields.

Despite the potential benefits of serious games in physical rehabilitation, many available platforms are inflexible and limited in their scope. Many developments do not follow a process involving a set of activities, actions, or tasks that must be executed when a software product is to be created. As a result, essential elements to the patient’s improvement process are ignored within the video game. Some of these elements are motivation, play levels, player commitment, challenges according to the patient’s level, clinical evaluation, assessment scales, among others [5,6].

This work aims to describe the software engineering frameworks used in serious games development and their benefits in the physical rehabilitation process.

### Background

#### A Note on Frameworks

The term *framework* has several meanings depending on the field. For example, it may refer to a model, prescription, guidelines underlying a design and analysis, among others.

The concept of framework is widely used in the field of computer science. However, there is some confusion between the software engineering framework and the application framework. The former provides a skeletal abstraction of a solution to several problems that have some similarities. A software engineering framework will generally outline the steps or phases that must be followed in implementing a solution without getting into the details of what activities are done in each phase [7]. The goal is for developers to use the framework as a guide to creating software systems by applying “building blocks” depending on the problem domain; by contrast, application framework is an integrated set of software artifacts (such as classes, objects, and components) that collaborate to provide a reusable architecture for a family of related applications [8]. They are used to facilitate the development process of applications, reducing time, effort, and costs.

Software engineering framework and application framework should not be confused. The latter is composed of pre-established source codes (eg, data access routines, form validation, templates) that the programmer uses to reduce workload and do not start the project from scratch.

One of the main motivations for applying a software engineering framework in serious game development is to design an efficient and satisfactory system for the patient.

#### Software Engineering Frameworks and Serious Games

The use of software engineering frameworks for the development of serious games allows the application of a variety of concepts, models, techniques, and artifacts at a high level of abstraction. Being an interdisciplinary field, an orientation on the developed tasks is required. Besides, it is flexible to adapt to changing conditions or personalization according to the final approach of the video game (rehabilitation, education, etc.).

Serious games like other software developments require a “systematic, disciplined, and quantifiable” approach. Every aspect of production, from early stages of system specification to maintenance after its operation, must be established. Below is a set of related activities that lead to the development of a software product [9-12].

#### Structural Activities in Software Development

In software engineering, 5 generic structural activities are used during software development [9-12]: communication, planning, modeling, construction, and deployment. The software process details will be different in each case, but the structural activities are the same. The definitions of the structural activities are presented in Textbox 1.

**Definitions of the structural activities in software development.**

**Communication**

Defining the software characteristics and functions is particularly important to communicate and collaborate with the client and other participants. This activity aims to understand the project objectives of the participants and meet the requirements.

**Planning**

Once the requirements are obtained, this activity presents an estimate of the resources; establishes a software project plan; and describes technical tasks, probable risks, and program activities.

**Modeling**

Its objective is to help understand the requirements through models. The models’ aim is to affirm the understanding of the work and give technical guidance to those who will implement the software, establishing, for example, the database model, the software architecture, user screen prototypes, and others. In some developments, this activity is the equivalent of the design stage.

**Construction**

This activity consists of the code generation and tests required to discover bugs in the software product.

**Deployment**

Once the software is created (completely or an increment), it is delivered to the client who will evaluate it and give feedback for system improvement.

#### Gamification

According to Kumar [13] gamification applies game design principles and mechanics to nongame environments. In the rehabilitation process, gamification can increase motivation and engagement through rewards, game levels, accessibility, feedback, and challenge. Therefore, the software engineering framework for serious game development must incorporate gamification. Various gamification elements include immersion, support for different roles, flow enhancement, visual enhancement, support for different learning stages and experience levels, design for interactivity, and progress [14]. By contrast, Vermeir et al [15] identified the following elements: avatar, challenge, competition, difficulty adjustment, feedback loops, levels, progress, rewards, social interaction, sound effects, and story/theme.

#### Benefits of Gamification in Rehabilitation

de Castro-Cros et al [16] analyzed the effects of gamification on the mental imagery brain–computer interface in rehabilitation functional assessments in 10 patients with stroke with hemiparesis in the upper limb and 6 healthy individuals. The authors concluded that user opinions about the game level of entertainment, clarity of rules, narrative, and visual attractiveness were all positive. The patients were consensus about the interest in gamifying stroke rehabilitation sessions. By contrast, Steiner et al [17] performed a scoping review of gamification in the rehabilitation of patients with musculoskeletal disorders of the shoulder. They concluded that gamification is essential in health care to enhance motivation and support therapy in general, especially in chronic diseases and rehabilitation. Other advantages are motivation, avoiding boredom, and distraction from pain and anxiety.

### Related Works

A systematic review of literature is a method to identify, evaluate, and interpret all available and relevant research of a particular research question, subject area, or phenomenon of interest. The individual studies that contribute to the systematic review are called primary studies. A systematic review is also considered a form of secondary study [18].

This systematic review includes literature work on developing serious games in physical rehabilitation using a software engineering framework. To identify existing secondary research in the same field, we searched the following electronic databases: IEEE Xplore, ACM Digital Library, Wiley Digital Library, PubMed, ScienceDirect, Taylor & Francis, Mary Ann Liebert, and Springer. Besides, we used Google Scholar as a web source to broaden our results.

The search was realized using the following search string: A1 AND B1 AND (C1 OR C2 OR C3 OR C4 OR C5 OR C6). Textbox 2 shows the terms included in the search string.

**Search terms to identify related secondary studies.**

**A term**

- A1. Serious games

**B term**

- B1. Framework

**C term**

- C1. Review
- C2. Systematic review
- C3. Systematic literature review
- C4. Systematic mapping
- C5. Mapping study
- C6. Systematic mapping study

When this search was performed in the electronic databases, no related secondary studies were identified. Therefore, we sought systematic reviews focused on software engineering frameworks in any field. Table 1 summarizes the secondary studies found.

Mubin et al [19] performed a review on gamification design framework and its application for children with autism. This review aimed to offer gamification solutions for interaction skills. They identified the framework phases in 5 papers and target users/audience/focus. The authors concluded that frameworks have been analyzed from an in-game context but did not emphasize on children with autism. In the literature, studies show that gamification is very effective in the areas of therapy and education for children with autism. The most important contribution of this review is the development of interaction skills. This review identified phases of the development process in some studies (eg, planning, designing). However, it does not explain how users benefit from the process interaction.

Vargas et al [20] developed a systematic mapping study on serious game quality. The aim was to discover the current state of serious games quality initiatives. One of the research questions focused on discovering if quality has been constant throughout the software development cycle or in some stages. The authors showed that 97% of the literature reviewed applied quality in the final phase (product). Only 7.14% focused on quality in the design phase and 1.79% in the requirement phase. This study was included because it identified the phases in which quality was applied: requirement, design, code, and final product.

Tomalá-Gonzáles et al [21] reported on methodologies, game engines currently used in serious games development in various areas (education, cognitive disabilities, and physical rehabilitation), and criteria for game engine selection. From the 27 papers, 8 used a defined methodology such as XP, Cascade, and others, while 3 proposed their own model. The authors concluded that although several software development methodologies can be adapted to serious game development, the best option was the SUM methodology because it is based on Scrum (fast, precise, optimized, and adaptable programming characteristics). However, this review did not make distinctions between framework and methodology. It also did not identify methodology phases nor the benefits of applying a methodology in the learning or rehabilitation process.

**Table 1:** Summary of secondary studies.

Study	Type	Year of publication	Target users/audience /focus	Benefit of framework	Phases of process development identified?
Mubin et al [19]	Review	2019	Children with autism	Interaction skills in children with autism	Yes
Vargas et al [20]	Systematic mapping study	2014	Serious games quality	Quality	Yes
Tomalá-Gonzáles et al [21]	Review	2020	Identifies methodologies and game engines	—^a^	No

^a^Not available.

Although our work shares similarities with the aforementioned studies, the literature review presented in this paper is different because this review (1) focuses on serious games for physical rehabilitation, (2) identifies the software development stages in each software engineering framework according to the structural activities proposed by Pressman [9], who states that “The software process details will be different in each case, but the structural activities are the same”; (3) identifies contributions of software engineering frameworks to the rehabilitation process; and (4) identifies if the proposed software engineering framework provides objective monitoring of the rehabilitation process.

## Methods

### Research Methodology

**Figure 1:**
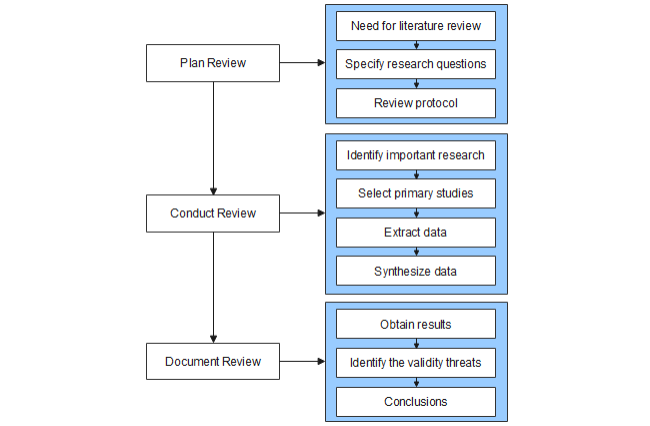
Literature review process.

The systematic literature review process proposed by Brereton et al [22] was applied for this systematic review. Figure 1 shows the process and steps for each phase. The process consists of 3 main phases: plan review, conduct review, and document review. The first phase consists of the following steps: (1) describe the main reasons for the literature review, (2) specify a set of research questions, and (3) review the protocol. The second phase comprises 4 steps: (1) identify important research, (2) select primary studies, (3) extract data from primary studies, and (4) synthesize data. Finally, the third phase consists of 3 steps: (1) obtain results, (2) identify the validity threats, and (3) conclusions. Figure 1 shows the literature review process. In the following subsections, we describe the activities carried out in each phase of this systematic literature review.

### Research Questions

In this subsection, we present the 9 research questions that guided this study through the investigation to meet the objectives of the systematic review. Table 2 presents these questions.

The research questions can be classified into 4 fields of interest. RQ1 and RQ2 study serious games evaluated in software engineering. These questions identify the number of serious games developed with a software engineering framework and the set of activities, actions, and tasks required.

RQ3 and RQ4 describe framework contributions to the rehabilitation process and implementation of gamification elements. It allows transforming obstacles into positive and fun reinforcements, thereby encouraging patients.

RQ5 and RQ6 are centered on applicability and serious game characteristics for rehabilitation using a software engineering framework. These questions identify relevant data such as target audience, interaction technology for data acquisition, main modalities, among others.

Finally, RQ7, RQ8, and RQ9 studied important aspects to evaluate and provide follow-up of rehabilitation progress depending on the type of exercise.

**Table 2:** Research questions.

Research question	Question
1	What framework is used in the development of the serious game?
2	What are the generic structural activities used in frameworks?
3	How the framework contributes to the rehabilitation process?
4	What gamification elements does the framework use?
5	What is the targeted disability contemplated in the frameworks?
6	If the framework includes a case study, which part of the body is rehabilitated? What is the modality of the serious game? Which interaction technology is used?
7	What type of evaluation and number of patients are involved in the clinical trials?
8	Does the framework contemplate a standardized scale to evaluate the patient’s rehabilitation progress?
9	Does the framework contemplate adaptability?

### Search Strategy

The objective of the search strategy was to identify all relevant primary studies. A literature search was conducted to answer the proposed research questions.

The search strategy is an adaptation of Guidelines for Performing Systematic Literature Reviews in Software Engineering [18] and Preferred Reporting Items for Systematic Reviews and Meta-Analyses (PRISMA) [23]. Relevant papers were identified by searching in the following databases: PubMed, PEDro (Physiotherapy Evidence Database), IEEE Xplore, ScienceDirect, ACM Digital Library, Mary Ann Liebert, Taylor & Francis Online, Wiley Online Library, and Springer. To build the search string, a list of keywords and their synonyms were identified. Logical operators (AND and OR) and words related to rehabilitation, serious games, and framework were used. The final search strings consisted of the following Boolean expressions: “(A1 AND (B1 OR B2)) AND (C1 OR C2 OR C3) AND D1”. The search terms are shown in Textbox 3.

**Search terms for the final search string.**

**A term**

- A1. Serious

**B term**

- B1. Game
- B2. Games

**C term**

- C1. Rehabilitation
- C2. Disability
- C3. Disabilities

**D term**

- D1. Framework

### Inclusion Criteria

The systematic review is focused on serious games for physical rehabilitation; clear inclusion criteria were established to determine the eligibility of papers for inclusion in the review. Only studies with the following criteria were considered eligible for inclusion: serious game papers for physical rehabilitation, papers published in English, and all serious games regardless of the year of development.

### Exclusion Criteria

Papers duplicated, papers regarding opinion pieces, existing literature reviews, papers that are not related to rehabilitation using serious games, serious games for educational purposes, and serious games for cognitive rehabilitation were excluded from the study.

### Study Selection

**Figure 2:**
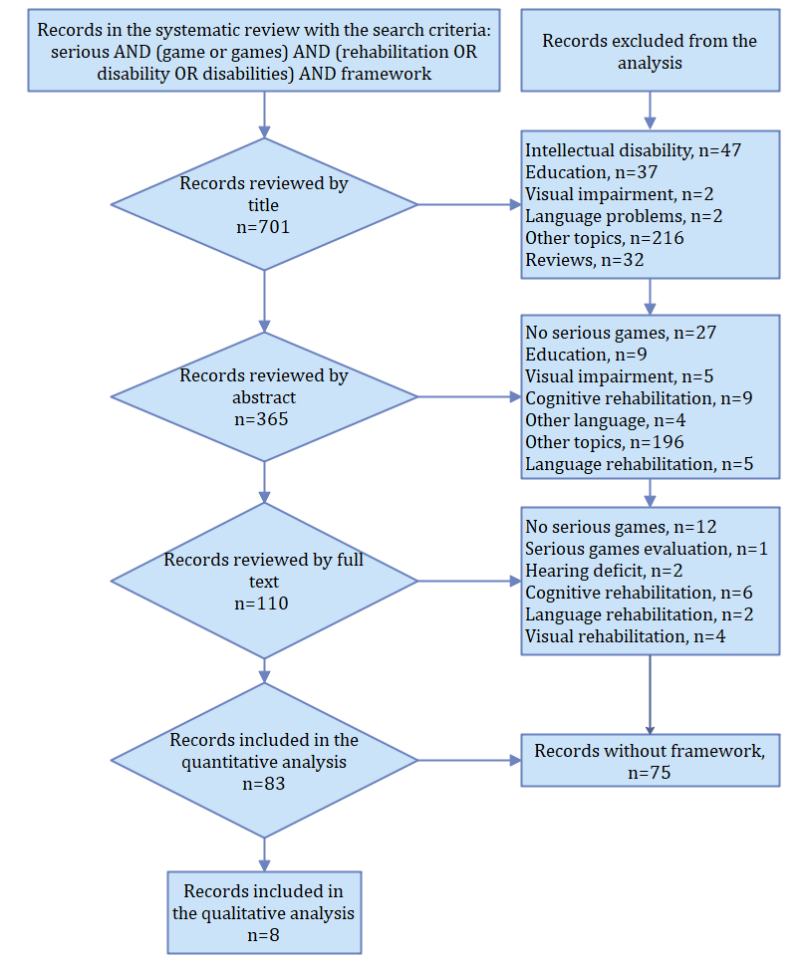
PRISMA (Preferred Reporting Items for Systematic Reviews and Meta-Analyses)–based flowchart.

First, the search string was used in different databases. Potentially relevant papers were identified after reading the title and abstract. Duplicate papers were removed. Subsequently, an exhaustive verification of compliance with the inclusion and exclusion criteria was carried out to select the papers. Figure 2 shows the item selection process. In the systematic review, 701 papers were included. Table 3 shows the number of documents retrieved from each database.

**Table 3:** Search results.

Databases	Results, n
PubMed	14
PEDro	12
IEEE Xplore	103
ScienceDirect	88
ACM Digital Library	166
Mary Ann Liebert	27
Taylor & Francis Online	50
Wiley Online Library	43
Springer	198

### Extract Data From Primary Studies

After identification, the primary papers were rigorously analyzed in accordance with the following considerations: (1) only the authors of this review can participate in the data collection process; (2) each primary paper should be reviewed with at least two reviewers; (3) each reviewer will collect a set of data from each primary study, then meet with another reviewer to reach an agreement on the data obtained.

Two types of data were extracted for each study: bibliographic (title, author name, country, year, database) and content data, which are used to answer the research questions. Table 4 shows the concentration of the bibliographic data of the primary papers.

Multimedia Appendix 1 shows the percentage of primary studies from each electronic database. IEEE Xplore presented more primary studies than the rest. The following section presents an analysis of the data collected.

**Table 4:** Bibliographic data of the primary papers.

Study	Year	Country	Database
Baranyi et al [24]	2013	Austria	IEEE Xplore
Pirovano et al [25]	2016	Italy	ScienceDirect
Amengual Alcover et al [26]	2018	Spain	PubMed
Baranyi et al [27]	2019	Austria	IEEE Xplore
Zain et al [28]	2012	Malaysia	IEEE Xplore
Noveletto et al [29]	2018	Brazil	ScienceDirect
Afyouni et al [30]	2017	Cyprus	ACM Digital Library
Maggiorini et al [31]	2012	Switzerland	ACM Digital Library

## Results

### RQ1: What Framework Is Used in the Development of the Serious Game?

Only 8 (10%) out of the 83 papers related to physical rehabilitation using a software engineering framework (Multimedia Appendix 2).

In Baranyi et al [24,27], the proposed studies were based on the user-centered design framework. The physiotherapist is important because s/he identifies the needs and limitations of the patients in the rehabilitation process. There are 3 phases: research, design, and evaluation. In research, a physiotherapist conducts brainstorming with the work team and identifies the requirements. Afterward, in the design phase, the team creates mock-ups and a prototype. Finally, the physiotherapist evaluates the application.

Pirovano et al [25] proposed a 4-step procedure to create safe exergames for rehabilitation therapies: exercise definition, virtualization, game design, and secondary goals. In exercise definition, a set of exercises is proposed to cover therapy needs. Each exercise is structured into primary and secondary goals. During virtualization, the team identifies primary goals, and they are implemented into a virtual exercise by defining input (tracking) and output (feedback) requirements through simple graphical elements and specifying interaction mechanisms. Through game design, the virtual exercise is converted into a true exergame. In the last step, there are 2 functionalities. The first is to analyze motion data and identify wrong movements. The second provides feedback to the patients.

In Amengual Alcover et al [26], the serious game development framework follows an iterative process flow structured into 2 dimensions: activities and incremental development. The first dimension is based on 3 approaches: Scrum, the web application development model, and a clinical trial. The activities dimension includes a project initiation activity, an iterative flow composed of 4 basic development activities (planning and control, modeling, construction, and evaluation), and a final clinical study to evaluate the rehabilitation process of the patient through the serious game. Incremental development includes 3 different increments: interaction mechanism, interaction elements, and serious game. In the first increment, an existing device on the market is identified to capture the movements of patients according to their needs. In the second increment, the development team must design the interaction elements that force patients to perform the therapy correctly. The final increment is aimed at designing a serious game that motivates the patient to perform therapy to obtain the best results.

Zain et al [28] proposed a conceptual framework for people with motor impairment, so they can enjoy the experience of playing serious games. The framework’s main elements were player skills, challenge, concentration, feedback, immersion, learning opportunities, accessibility, and adaptivity. The proposed framework will help the game designer and developer create a serious game that combines the game’s technology with the learning environment. This framework is based on the game flow model.

Noveletto et al [29] presented a conceptual model for the design or development of serious games to rehabilitate people with stroke. The framework establishes a relationship between experts and patients to obtain the requirements, considering that the biomedical device and the video game score are used to design serious games.

Afyouni et al [30] proposed a framework consisting of a therapy-driven 3D environment augmented with a natural user interface based on movement. The framework incorporated different adaptation techniques to adjust patient’s needs. Patient preferences and limitations were considered key parameters for changing the game, thereby creating personalized games for each patient.

Maggiorini et al [31] presented a framework for serious game development that allows the therapist to remotely control the video game home activities. The objective was to create a more attractive game for the elderly with easily adjustable parameters for therapy adaptability. The framework includes 3 phases of serious game development: requirements definition, empirical validation of requirements list, and design and prototyping.

### RQ2: What Are the Generic Structural Activities Used in Frameworks?

The objective of this research question was to identify generic structural activities in primary studies (see the “Background” section). Table 5 summarizes the structural activities and Multimedia Appendix 3 shows the frequency of occurrence of each structural activity in primary studies.

Every study established a communication activity to obtain the requirements. Baranyi et al [24] brainstormed with a physiotherapist to identify relevant problems and needs for patients undergoing rehabilitation. Pirovano et al [25] defined exercises addressing the primary and secondary objectives of rehabilitation. To achieve maximum effectiveness, the exercises are defined in collaboration with therapists. In Amengual Alcover et al [26], the communication began by identifying the context, operational objectives, restrictions, and requirements. Baranyi et al [27] established communication with the therapist to obtain the requirements. Zain et al [28] identified the user abilities, limitations, and behavior, which become requirements for the serious game. Noveletto et al [29] considered experts in the field (health personnel, therapists, etc.) and patients to obtain the requirements. Afyouni et al [30] established the type of game through patient needs, preferences, and limitations, allowing custom game features. Finally, Maggiorini et al [31] analyzed the most diffused issues present in elders’ homes (eg, size of rooms, habits) to explore requirements and limitations through an immersive approach.

The planning activity was implemented in Amengual Alcover et al [26]. The goal of this activity was to determine the tasks to perform during the development by identifying the end products and the people who will do the work. The activity includes 3 tasks: planning, scheduling, and tracking.

The modeling activity was performed in several papers. For example, Baranyi et al [24] called it design, elaborating basic models discussed with a therapist. Pirovano et al [25] transformed the exercise requirements into a true exergame by adding all the elements and characteristics of a game and a good game design for the patients. Amengual Alcover et al [26] created models that helped the development team to understand the requirements obtained and the game design. By contrast, Baranyi et al [27] contemplated the use of prototypes to refine user requirements. Finally, Maggiorini et al [31] established a list of technical characteristics (desired) for the prototype creation.

The construction activity was implemented in every study. Developments produce executable software units that will be used by users, through the creation of prototypes to improve the software [24-27,30,31], or the final product [28,29].

Finally, the user evaluates and provides feedback on the serious game in the deployment stage. In the primary papers, Pirovano et al [25] and Baranyi et al [27], patients were asked to give their opinion to improve the game design and change some aspects of the application.

**Table 5:** Structural activities in primary studies.

Study	Communication	Planning	Modeling	Construction	Deploy
Baranyi et al [24]	X	—^a^	X	X	—
Pirovano et al [25]	X	—	X	X	X
Amengual Alcover et al [26]	X	X	X	X	—
Baranyi et al [27]	X	—	X	X	X
Zain et al [28]	X	—	—	X	—
Noveletto et al [29]	X	—	—	X	—
Afyouni et al [30]	X	—	—	X	—
Maggiorini et al [31]	X	—	X	X	—

^a^Not available.

### RQ3: How the Framework Contributes to the Rehabilitation Process?

Baranyi et al [24,27] applied a user-centered design approach to establish constant communication with the physiotherapist who has the experience to identify the needs and limitations of the final user. Serious games are developed with entertainment elements such as levels, rewards, challenges, and adaptability to the patient need, considering special conditions.

Pirovano et al [25] proposed the creation of safe exergames, identifying the needs of real exercise besides therapy goals. These needs are incorporated into a video game considering the primary objectives (what a user should do) and secondary objectives (how user actions should be carried out). The former is easily integrated into the gameplay, while the latter aids the patient with corrections or prevention of compensatory movement through analysis of the flow of movement data and wrong movements in real time, thereby providing immediate feedback to patients to correct themselves during the exercise.

Amengual Alcover et al [26] proposed an iterative, prototype-oriented, systematized serious game development process. The proposed process guarantees that products based on this framework are developed and validated by following a coherent and systematic method that leads to high-quality serious games.

For users with motor impairment, Zain et al [28] used flow theory [32] to propose user interface design factors that make their experience enjoyable when playing serious games. This framework includes user interface design factors and aims to establish a conceptual model that can be used by a game designer for efficient game development or an educational practitioner when designing enjoyable serious games for users with motor impairment.

Noveletto et al [29] established a relationship among key stakeholders (experts and patient) and elements (biomedical device and game score) for serious game design. The framework states that a correlation between the game score and clinical tests can aid treatment and evaluation through the biomedical system.

Afyouni et al [30] proposed a framework for video game development with an adaptive and user-centered approach. The framework embeds different adaptation techniques to tailor to patients’ needs. The video game adapts to the difficulty level based on the patient’s profile and performance in real time. Other aspects such as patient preferences and constraints are considered as key game-changing parameters.

Finally, in Maggiorini et al [31], the framework allowed serious game development with telerehabilitation allowing the therapist to remotely control the video game home activities. It supports parameter adjustments for therapy adaptability. Table 6 summarizes framework contributions.

**Table 6:** Framework contributions in primary studies.

Framework contribution to rehabilitation	Utility	Primary studies
Communication with health expert	A physiotherapist establishes communications with patients undergoing rehabilitation to identify the problems and needs.	Baranyi et al [24], Pirovano et al [25], Baranyi et al [27], Noveletto et al [29]
Exercise definition	Exercise can be defined as a sequence of different actions needed to complete it to achieve maximum effectiveness.	Pirovano et al [25]
Analyzes the stream of motion data and identifies in real time wrong movements	Provides immediate feedback to the patients for correct exercising.	Pirovano et al [25]
Iterative and prototyping	Visualize prototypes of serious games from early stages. The therapist or patients identify additional requirements or modify them.	Baranyi et al [24], Pirovano et al [25], Baranyi et al [27], Noveletto et al [29], Afyouni et al [30], Maggiorini et al [31]
User interface design factors	Motivation and immersion	Baranyi et al [24], Pirovano et al [25], Amengual Alcover et al [26], Baranyi et al [27], Zain et al [28], Noveletto et al [29]
The correlation between game score and clinical tests	Aids in patient treatment and evaluation	Noveletto et al [29]
Adaptive approach	Adapts difficulty level according to the patient’s profile and performance in real time	Baranyi et al [24], Pirovano et al [25], Zain et al [28] Afyouni et al [30], Maggiorini et al [31]
Telerehabilitation	Therapists can remotely control the video game for home activities and provide adjustable parameters to improve therapy	Maggiorini et al [31]

### RQ4: What Gamification Elements Does the Framework Use?

#### Overview

Gamification allows the transformation of obstacles into positive and fun reinforcement, encouraging users to make the right decisions for their health and well-being [33]. It is essential to keep the patient motivated in physical rehabilitation. For this reason, the software engineering framework is required to use gamification elements. The papers identified the following elements: feedback, motivational factor, adaptability, challenge, levels, immersion, rewards, concentration, and avatar. Table 7 shows the gamification elements in primary studies, and Multimedia Appendix 4 shows the frequency of occurrence of each gamification element.

The gamification elements of primary studies are described below.

**Table 7:** Gamification elements in primary studies.

Gamification element	Study
Feedback	Pirovano et al [25], Amengual Alcover et al [26], Baranyi et al [27], Zain et al [28], Noveletto et al [29], Afyouni et al [30], Maggiorini et al [31]
Motivational factor	Baranyi et al [24], Pirovano et al [25], Amengual Alcover et al [26], Baranyi et al [27], Noveletto et al [29]
Adaptability	Baranyi et al [24], Pirovano et al [25], Zain et al [28], Afyouni et al [30], Maggiorini et al [31]
Challenge	Baranyi et al [24], Zain et al [28], Afyouni et al [30]
Levels	Baranyi et al [24], Amengual Alcover et al [26], Baranyi et al [27], Afyouni et al [30]
Immersion	Zain et al [28]
Rewards	Pirovano et al [25]
Concentration	Zain et al [28]
Avatar	Pirovano et al [25]

#### Feedback

In Pirovano et al [25], the feedback mechanisms were designed to show the outcome of actions to patients. For instance, whether a target is met or a movement has been successfully performed. Amengual Alcover et al [26] used “mirror feedback,” which consists of projecting the user onto the screen and simulating a mirror in such a way that the users can see themselves on the screen at all times. In Baranyi et al [27], the feedback provided was either visual, aural, or haptic. In Zain et al [28], users with motor impairment received feedback on their progress, and when they lose the game, feedback is provided to continue in the right direction. Noveletto et al [29] established that serious same should reward players with feedback on progress. Afyouni et al [30] used a scoring system that was designed to keep track of the number of times the patient successfully passed through the targets. Finally, in Maggiorini et al [31], a skeleton wireframe is drawn in red to provide immediate visual feedback, and an alarm is raised on the screen.

#### Motivational Factor

Baranyi et al [24] used “goals.” The gameplay was based on achieving goals that should act as motivation factors. Pirovano et al [25] established that extrinsic motivational effects can be achieved through careful use of verbal praise, scoring mechanisms, and virtual reward systems. In Amengual Alcover et al [26], the development of new serious games allowed the inclusion of motivational elements to increase engagement. Baranyi et al [27] used rewards in serious games for the user. Finally, Noveletto et al [29] used the “motivational score” to improve attention during rehabilitation sessions.

#### Adaptability

Baranyi et al [24] proposed an adaptive system with the opportunity to adapt the game difficulty. Pirovano et al [25] established that virtual exercises should use dynamic difficulty adaptation, thus further increasing the flexibility of serious games. For Zain et al [28], an adaptive factor was important to design and develop serious games for users with motor impairment because the application, aware of the users’ current cognitive load and physical limitations, can change its response, presentation, and interaction flow to improve users’ experience and their task performance. In Afyouni et al [30], the framework embeds different adaptation techniques to adapt to the patients’ needs. Key game-changing parameters such as patient preferences and constraints are considered. This allows the creation of personalized game features for every patient. Maggiorini et al [31] proposed that remotely controlled serious games may also provide easily tunable parameters to better adapt the game therapy to the actual patient recovery.

#### Challenge

Baranyi et al [24] proposed the challenge as a “key fact.” They considered that the game should not be too easy nor too hard to manage. The game should be sufficiently challenging and match the player’s skill level. Zain et al [28] proposed that serious games should also vary the level of difficulty and keep an appropriate pace. Afyouni et al [30] generated therapy-aware navigational movements with multiple levels of difficulty.

#### Levels

Baranyi et al [24] stated that the purpose of the serious game developed is to have a rehabilitation system containing different levels that were adapted and created for the individual needs of the patients and to fit their impairments. Amengual Alcover et al [26] considered that serious games must have a definition of different levels in the game. In Baranyi et al [28], when the game is started for the first time, a diagnostic routine is performed; using these data, a baseline for the exercises can be defined by the therapist to get an initial idea about how easy or complex a level might be for a patient. Afyouni et al [30] presented different levels of difficulty based on therapeutic gestures and patient performance.

#### Immersion

Zain et al [28] considered that immersive games draw players into the game and affect their senses through elements such as audio and narrative.

#### Reward

Pirovano et al [25] used a scoring system, and at the end of each exergame, a virtual reward is presented to the patients.

#### Concentration

Zain et al [28] considered that the more concentration a task requires in terms of attention and workload, the more absorbing it will be. The games should grab the player’s attention quickly and maintain it throughout the game.

#### Avatar

Pirovano et al [25] used an avatar for feedback on wrong movements, changing the color of the associated avatar segments. When wrong movements persist for a long time, the game is paused, and a virtual therapist avatar pops up to advise patients.

### RQ5: What Is the Targeted Disability Contemplated in the Frameworks?

This specifies whether a study focuses on a particular pathology with loss or decrease in movement. The papers established the following target pathology: 4 defined strokes [24,25,27,29], 2 defined neuromotor disorder [26,30], 1 defined users with motor impairment [28], and 1 defined rehabilitation of the elderly [31]. Stroke is mainly targeted in studies because it is the second cause of death and the third cause of disability worldwide [34]. Multimedia Appendix 5 shows the target disability percentage.

### RQ6: If the Framework Includes a Case Study, Which Part of the Body Is Rehabilitated? What Is the Modality of the Serious Game? Which Interaction Technology Is Used?

As Table 8 reports, Baranyi et al [24] presented a prototype that rehabilitates patients with lower limb disabilities with balance and strength exercises using the Wii Fit Balance Board. Pirovano et al [25] developed serious games for upper limb motor rehabilitation therapy using Microsoft Kinect and lower limb with the Wii Fit Balance Board. Amengual Alcover et al [26] also rehabilitated the lower limb by allowing patients to perform repetitions in a video game controlled with Microsoft Kinect, with each repetition varied according to the participant’s tolerance and the physiotherapist’s recommendations. Baranyi et al [27] performed hand rehabilitation using gesture exercises, touch, and patient movement levels using mobile phone sensors. Zain et al [28] and Noveletto et al [29] did not report any case studies. Afyouni et al [30] developed a serious game for hand rehabilitation using leap motion. Game instructions can be visual (shown on the screen) or voice, depending on the perception capacity of the patient. Finally, Maggiorini et al [31] developed a prototype for rehabilitation using Microsoft Kinect. It only presents the skeleton tracking by a sensor and does not mention whether the video game implements another form of communication with the patient.

The modality is a way in which information is transmitted from the computer to the participants [35]. Baranyi et al [24,27], Pirovano et al [25], Amengual Alcover et al [26], Afyouni et al [30], and Maggiorini et al [31] used a visual modality, presenting a graphical interface for user interaction. Pirovano et al [25], Amengual Alcover et al [26], Baranyi et al [27], and Afyouni et al [30] used audio effects such as music or voice instructions. Baranyi et al [27] used haptic modality to control the video game through a touch screen. Zain et al [28] and Noveletto et al [29] did not report modalities.

**Table 8:** Rehabilitated limb, serious game modality, and data-acquisition device in primary studies.

Study	Rehabilitation/extremity	Modality	Interaction technology
Baranyi et al [24]	Lower limbs	Visual	Wii Fit Balance Board
Pirovano et al [25]	Lower and upper limbs	Visual, auditory	Wii Fit Balance Board and Microsoft Kinect
Amengual Alcover et al [26]	Lower limbs	Visual, auditory	Microsoft Kinect
Baranyi et al [27]	Hand	Visual, auditory, haptic	iOS platform sensors
Zain et al [28]	Not reported	Not reported	Open
Noveletto et al [29]	Not reported	Not reported	Open
Afyouni et al [30]	Hand	Visual, auditory	Leap motion
Maggiorini et al [31]	Full body	Visual	Microsoft Kinect

### RQ7: What Type of Evaluation and Number of Patients Are Involved in the Clinical Trials?

The objective of this research question was to identify clinical validation of the studies and the number of patients involved. In clinical trials, participants receive specific interventions according to the research plan or protocol created by the researchers to determine the safety and efficacy of the interventions through the measurements of the outcomes [36]. Table 9 shows these data. Amengual Alcover et al [26] conducted a clinical trial and observed a significant difference between before and after scores. They used the Berg Balance Scale and their results showed a significant functional improvement (P=.002) in comparison with assessments before (mean 29.5 [SD 3.9] and after (mean 34.1 [SD 2.2]) the intervention. The Functional Reach Test revealed significant differences in functional balance before and after the intervention: right upper limb, before (mean 8.6 [SD 1.4]) and after intervention (mean 10.1 [SD 2.0]; P=.007); and left upper limb, before (mean 8.3 [SD 2.0]) and after intervention (mean 10.1 [SD 3.7]; P=.052). Finally, a significant difference between the pre- and post-assessment scores for the Tinetti Balance Test was observed at the end of the 24-week intervention period. The average score rose from 16 to 21 points on a scale of 28 points. Afyouni et al [30] reported that patients showed improved hand movement using a range of motion. They were able to document 66% of the elements in the video game. No other study reported a clinical trial.

**Table 9:** Type of evaluation and number of patients in the primary studies.

Study	Evaluation	Number of patients
Baranyi et al [24]	No clinical validation	N/A^a^
Pirovano et al [25]	No clinical validation	N/A
Amengual Alcover et al [26]	Clinical trial	9
Baranyi et al [27]	No clinical validation	N/A
Zain et al [28]	No clinical validation	N/A
Noveletto et al [29]	No clinical validation	N/A
Afyouni et al [30]	Clinical trial	5
Maggiorini et al [31]	No clinical validation	N/A

^a^NA: not applicable.

### RQ8: Does the Framework Contemplate a Standardized Scale to Evaluate the Patient’s Rehabilitation Progress?

An assessment instrument allows to objectively quantify the disability degree of the patient and measure the progress of rehabilitation [37,38]. The evaluation scales in the framework are used to quantify the improvement in rehabilitation depending on the type of exercise applied. During the analysis of primary papers, we identified 3 studies with assessment instruments: Pirovano et al [25], Amengual Alcover et al [26], and Afyouni et al [30].

### RQ9: Does the Framework Contemplate Adaptability?

Adaptability is the ability to dynamically adapt difficulty in a video game according to the patient’s performance [39]. Five primary studies use this characteristic. In Baranyi et al [24], the physiotherapist designed the level of difficulty of the video game. Pirovano et al [25] established that for every exercise, quality parameters are necessary to define movement properties. This will allow one to determine the challenge degree of the exercises and adapt the difficulty to the patient’s needs. Zain et al [28] mentioned that adaptability must consider the following elements: (1) user motivation, (2) experience and abilities, and (3) detection, which identifies necessary changes. Afyouni et al [30] adapted the difficulty level based on the patient’s profile and performance in real time. In Maggiorini et al [31] the therapist can remotely adapt the game therapy to the patient’s actual recovery. Amengual Alcover et al [26], Baranyi et al [27], and Noveletto et al [29] did not specify how adaptability is incorporated into their game. Multimedia Appendix 6 shows the percentage of frameworks contemplating adaptability.

### Threats to Validity of Primary Studies Selected

Although we used search strategies and techniques to systematically find papers by using keywords in the selected databases, these words may vary within papers, so some relevant studies may have been omitted.

## Discussion

### Preliminary Findings

We found only few studies that used a systematic process for serious game development. Each framework analyzed in the primary papers highlighted a different feature.

Planning was the structural activity least implemented. This activity is essential because it allows goal definition, objectives, and path to follow in the software development [9,10,40,41].

Regarding applicability, most studies focused on the treatment of stroke sequelae using various modalities such as visual and auditory. The latter should also be implemented to provide feedback on patient performance. Lastly, test cases directly use playable commercial platforms such as Microsoft Kinect and Leap motion as interaction technology.

There were a few clinical trials, and the type of improvement reported varies from one study to another. Amengual Alcover et al [26] used the Berg Balance Scale and Tinetti Balance Test measurements and reported significant functional improvement from previous results. Afyouni et al [30] also reported improvements using range of motion evaluation in hand movement. No other studies used clinical trials to evaluate the framework. Clinical evaluation is essential to objectively validate the patient’s rehabilitation progress [36].

Pirovano et al [25], Amengual Alcover et al [26], and Afyouni et al [30] used an evaluation scale to assess the patient’s progress. It should also be used as an alternative to adaptability, which is essential for progress and motivation [42]. It is also a technique that can be used to advance game levels [5]. Game levels help engage in the game and could increase treatment compliance.

### Conclusions

The objective of this study was to identify the software engineering frameworks used in the development of serious games through a literature review of 8 primary studies. The conclusions of this study are as follows:

About 75% (6/8) of the primary papers proposed a framework [25,26,28-31], whereas the rest were adaptations of the user-centered design framework (RQ1). Regarding the structural activities, 100% (8/8) of the papers applied the communication and construction activity [24-31], 63% (5/8) used modeling (known as a design in some developments) [24-27,31], 25% (2/8) considered user feedback to improve the serious games [25,27], and only 13% (1/8) included the planning phase [26] (RQ2).

Each primary study contributes in one or more aspects to the rehabilitation process. Baranyi et al [24,27] applied a user-centered design using which the physiotherapist can personalize individual needs in the serious game. Pirovano et al [25] proposed ease of play and assisted help during the rehabilitation exercise. Amengual Alcover et al [26] developed a framework for motor rehabilitation therapies using a systematized process. Zain et al [28] embraced immersion and fun in the game to maintain flow interest. Noveletto et al [29] used game scores for patient assessment. Afyouni et al [30] developed games with dynamic adaptability that were patient centered. Finally, Maggiorini et al [31] incorporated telerehabilitation and adaptability for the elderly to perform rehabilitation exercises at home (RQ3). Every study applies gamification elements that allow patients to transform rehabilitation obstacles into positive and fun reinforcements. Feedback was the gamification element most applied (7/8, 88%) [25-31]. Other elements frequently implemented were adaptability [24,25,28,30,31] and motivational factor [24-27,29] (5/8, 63%) for both; RQ4.

Stroke is the primary pathology on which serious games are focused. This pathology is the third cause of disability worldwide, and a characteristic symptom is the sudden, generally unilateral, loss of muscle strength in the arms, legs, or face (RQ5). Regarding the case studies of limb rehabilitation, 2 studies [24,26] included the lower limb, 1 [25] included lower and upper limbs, 2 [27,30] included hand, 1 [31] full body, and 2 [28,29] did not report case studies. The most used video game modality was visual (6/8, 75%) [24-27,30,31], followed by auditory (4/8, 50%) [25-27,30]. Although each case study used a different motion acquisition technology, every framework allowed a wide variety of the interaction style to obtain the patient’s movement and control the serious game (RQ6).

Of the primary papers, 25% (2/8) applied a clinical evaluation to assess patient improvement when the serious game is used [26,30] (RQ7). To objectively evaluate progress and identify abilities and deficits, only 38% (3/8) of the primary studies used an assessment instrument [25,26,30] (RQ8). The assessment used standardized procedures indicating how a patient of any given age and intelligence level would perform. Adjusting the video game difficulty to the patient’s rehabilitation needs is essential to avoid frustration or boredom, and 63% (5/8) of the primary studies used adaptability [24,25,28,30,31] (RQ9).

Finally, we recommend that all serious games have to be developed with a framework or methodology. If for some reason this is not possible, they should at least involve the therapist to define requirements. It is also important to include evaluation scales to measure the patient’s progress and gamification elements. Besides, the video game development must be an iterative and incremental process based on generic structural activities and the patient should be considered in the validation and feedback phases.

We propose the following recommendations for future studies:

- Carry out a study of the papers that propose a methodology for serious game development.
- Study software engineering framework proposals in serious games from other fields, such as education.
- Develop a software engineering framework applying all the structural activities and gamification elements for the creation of serious games for physical rehabilitation.

## Appendix

Multimedia Appendix 1 Percentage of primary studies provided by each electronic database.

Multimedia Appendix 2 Percentage of serious games that used a software engineering framework.

Multimedia Appendix 3 Frequency of occurrence of structural activities in primary studies.

Multimedia Appendix 4 Frequency of occurrence of each gamification element.

Multimedia Appendix 5 Target disability contemplated in frameworks.

Multimedia Appendix 6 Frameworks contemplating adaptability.

## Abbreviations

PRISMA: Preferred Reporting Items for Systematic Reviews and Meta-Analyses
