# Supplementary figures and images for: Software Engineering Frameworks Used for Serious Games Development in Physical Rehabilitation: Systematic Review
Source: JMIR Serious Games. 2021 Nov 11;9(4):e25831. doi: 10.2196/25831 (PMC8663647; doi:10.2196/25831)

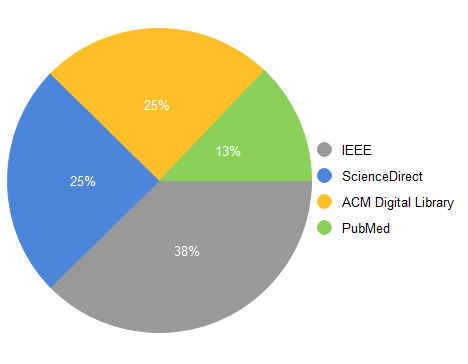

Supplement: Multimedia Appendix 1 [file games_v9i4e25831_app1.png]

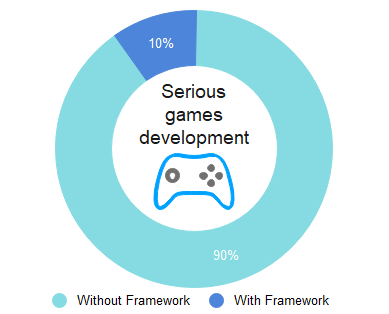

Supplement: Multimedia Appendix 2 [file games_v9i4e25831_app2.png]

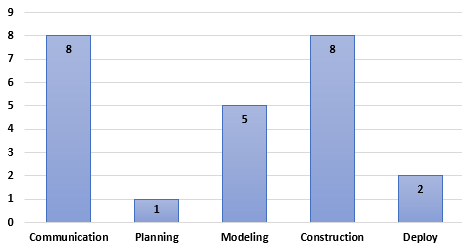

Supplement: Multimedia Appendix 3 [file games_v9i4e25831_app3.png]

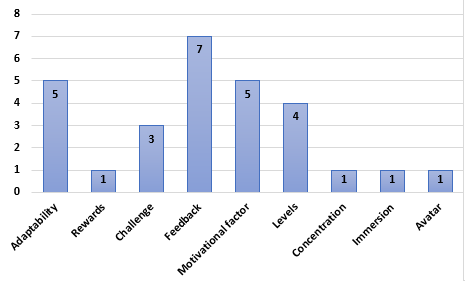

Supplement: Multimedia Appendix 4 [file games_v9i4e25831_app4.png]

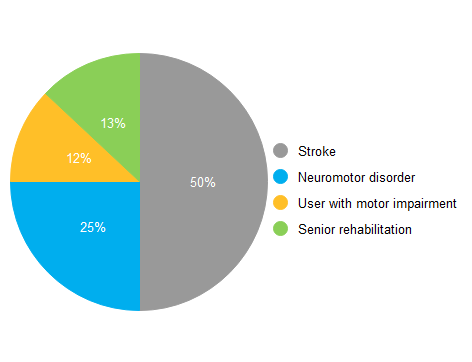

Supplement: Multimedia Appendix 5 [file games_v9i4e25831_app5.png]

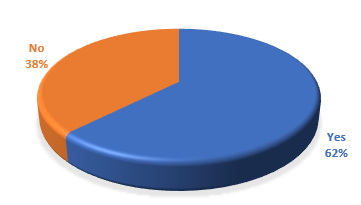

Supplement: Multimedia Appendix 6 [file games_v9i4e25831_app6.png]
